# Structure Matters: Asymmetric CO Oxidation at Rh Steps with Different Atomic Packing

**DOI:** 10.1021/jacs.2c06733

**Published:** 2022-08-12

**Authors:** Fernando García-Martínez, Lisa Rämisch, Khadiza Ali, Iradwikanari Waluyo, Rodrigo Castrillo Bodero, Sebastian Pfaff, Ignacio J. Villar-García, Andrew Leigh Walter, Adrian Hunt, Virginia Pérez-Dieste, Johan Zetterberg, Edvin Lundgren, Frederik Schiller, J. Enrique Ortega

**Affiliations:** † Departamento Física Aplicada, Universidad del País Vasco, San Sebastián 20018, Spain; ‡ Department of Physics, Lund University, Lund 221 000, Sweden; ¶ Centro de Física de Materiales CSIC/UPV-EHU-Materials Physics Center, Manuel Lardizábal 5, San Sebastián 20018, Spain; § National Synchrotron Light Source II, Brookhaven National Laboratory, Upton, New York 11973, United States; ∥ NAPP Station, CIRCE Beamline, ALBA synchrotron, Carrer de la Llum 2-26, Cerdanyola del Vallès 08290, Spain; ⊥ Donostia International Physics Centre, Manuel Lardizábal 4, San Sebastián 20018, Spain

## Abstract

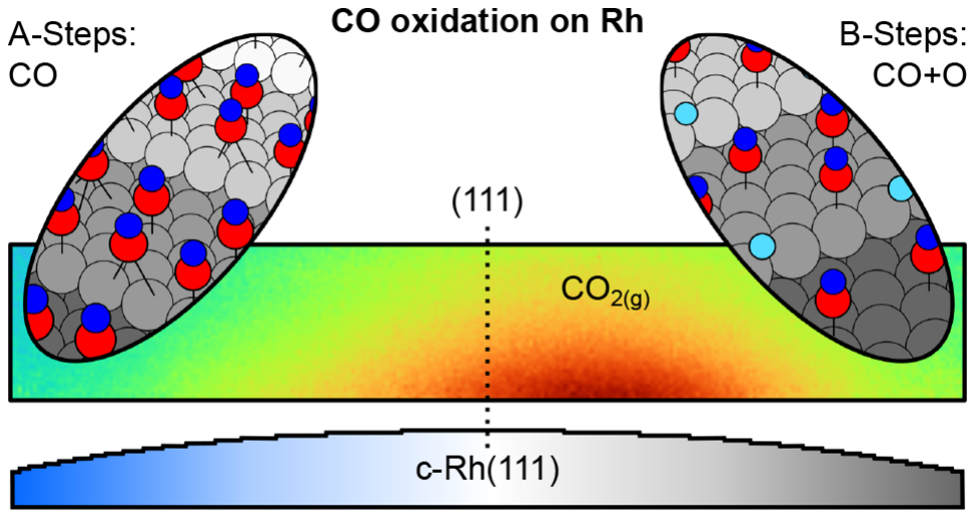

Curved crystals are a simple but powerful approach to bridge the gap between single crystal surfaces and nanoparticle catalysts, by allowing a rational assessment of the role of active step sites in gas-surface reactions. Using a curved Rh(111) crystal, here, we investigate the effect of A-type (square geometry) and B-type (triangular geometry) atomic packing of steps on the catalytic CO oxidation on Rh at millibar pressures. Imaging the crystal during reaction ignition with laser-induced CO_2_ fluorescence demonstrates a two-step process, where B-steps ignite at lower temperature than A-steps. Such fundamental dissimilarity is explained in ambient pressure X-ray photoemission (AP-XPS) experiments, which reveal partial CO desorption and oxygen buildup only at B-steps. AP-XPS also proves that A-B step asymmetries extend to the active stage: at A-steps, low-active O–Rh–O trilayers buildup immediately after ignition, while highly active chemisorbed O is the dominant species on B-type steps. We conclude that B-steps are more efficient than A-steps for the CO oxidation.

## Introduction

CO oxidation is a fundamental pillar for the understanding of surface catalytic reactions, and, accordingly, it is among the most studied reactions in surface science.^1−4^ However, the majority of scientific investigations have been performed with either single crystals or under ultrahigh vacuum, far from the operando requirements of industry. Therefore, an extrapolation of their conclusions to real catalytic systems (atmospheric pressures and powder/nanoparticle catalysts) may not be correct.^5^ On one side, metallic nanoparticles comprise several facets, and monitoring their specific activity and interplay during a chemical reaction is challenging.^6^ On the other side, the information that can be obtained using conventional flat crystals is restricted to one plane alone, and hence it is not representative of the structure of a real catalyst.^7^ One approach to partially close this structure gap is to use cylindrical sections of single crystals, since their curved surface provides a smooth variation of the crystal orientation that allows to systematically compare different facets under the very same reaction conditions.^8−11^

CO oxidation on Rh is characterized by two well-defined stages, depending on the catalyst temperature.^12^ At low temperature, adsorbed CO molecules block the O_2_ dissociative adsorption. Co-adsorption of reactants is not possible, and hence there is very low CO_2_ turnover.^13^ At higher temperatures, however, most of the CO molecules will desorb from the surface, leaving empty sites that are typically occupied by oxygen species. Co-adsorption of reactants is now possible, leading to a substantial CO_2_ production.^12^ The transition between the poisoned (CO-covered) and active (O-covered) stages occurs at the so-called light-off (or ignition) temperature *T*_i_. A detailed knowledge on the CO ignition at different crystal facets is mandatory to tailor new catalysts and improve the energy costs of industrially relevant processes.

The adsorption of both CO and O_2_ has been previously studied on various Rh surfaces with atomic-scale precision, which facilitates operando studies of the CO oxidation reaction with the same level of detail, such as the present one. At low temperature, well below the ignition, CO will adsorb in Rh(111) terraces in top and hollow positions,^14−16^ while it will anchor to top and bridge sites at the steps of A- and B-type surfaces.^17−19^ On the other hand, O_2_ adsorption on flat and stepped Rh surfaces will lead to dissociation into single oxygen atoms at face-centered cubic (fcc) hollow sites, eventually forming oxide stripes as the oxygen coverage increases.^20,21^ At higher O coverages, several works at different Rh facets and nanoparticles report the formation of the Rh surface oxide consisting of O–Rh–O (RhO_2_) trilayers.^20−27^ Such surface oxide is a less-efficient catalyst for the CO oxidation than the metallic surface covered with chemisorbed oxygen,^12,28^ possibly due to the fact that CO does not stick on the surface oxide and the reaction is restricted to oxide-metal boundaries.^20^

Here, we explore the evolution of chemical species during the thermal activation of the CO oxidation at A- and B-type vicinal surfaces simultaneously, using a curved Rh(111) crystal. Planar laser-induced fluorescence (PLIF) reveals an asymmetric light-off, where B-steps ignite at a lower temperature than A-steps. As judged by near ambient-pressure X-ray photoemission spectroscopy (NAP-XPS), such asymmetry is caused by the partial CO-depletion and O-accumulation exclusively at B-steps. During the active stage of the reaction, after the ignition of the entire sample, we observe surface oxide formation at the (111) terraces and A-steps, while the oxygen at B-steps remains chemisorbed. Therefore, since B-steps do not oxidize further during reaction conditions, we conclude that they are more active toward the CO oxidation than A-type steps.

## Experimental Section

### Curved Rh(111) Crystal

**Figure 1:**
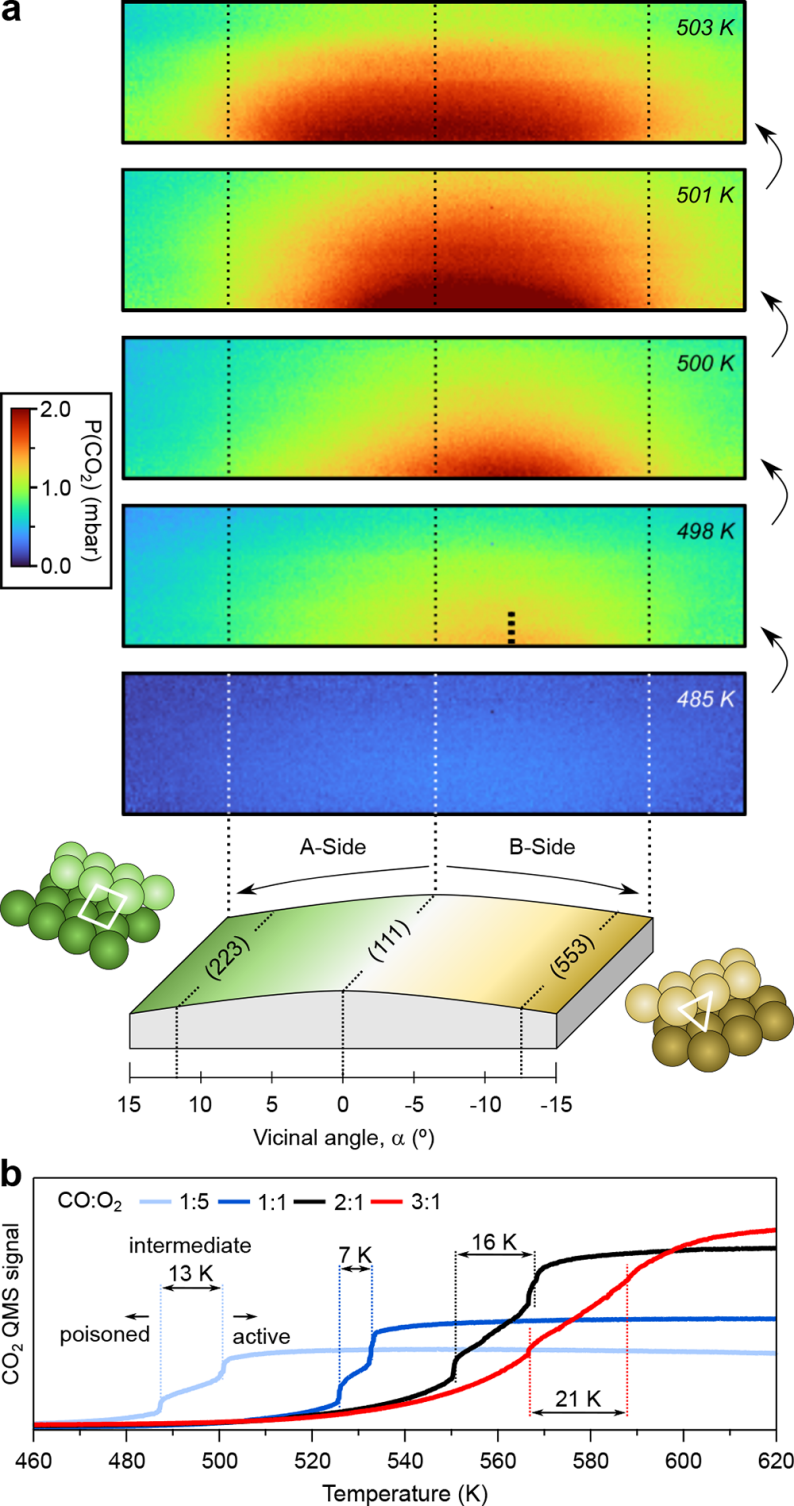
(a) PLIF images acquired under a 24 mbar, 1:5 CO:O_2_ gas mixture. Argon was added to reach 100 mL/min and 150 mbar of total flow and pressures, respectively. The average heating slope was 12 K/min. Individual snapshots during the activation process, from the poisoned stage (485 K, no CO_2_ turnover) to the active stage (503 K), are shown. The sample design is illustrated in the bottom, together with the position of relevant surfaces across the curved crystal and the vicinal angle α scale (i.e., step density^38^). (b) CO_2_ signal in the QMS (mass 44), as a function of the temperature for individual experiments varying the CO:O_2_ pressure ratio. Vertical lines mark the two abrupt ignition steps that define the entire activation process. Movies of the ignition at CO:O_2_ pressure ratios of 1:5 (Figure 1a), 1:1, and 2:1 are provided in Supplementary Movies 1, 2, and 3.

The c-Rh(111) sample (see bottom of Figure 1a) possesses the (111) plane at the apex of the crystal, and its cylinder axis is parallel to the [11̅0] direction. This leads to surfaces with a smooth increase of either A-type (100 microfacet) or B-type (111 microfacet) close-packed steps as one departs from the center of the crystal.^9,10,19,29^ Vicinal angles (α), directly related to the step density,^19^ up to α = ± 15°, can be probed using this sample. This allows to reach vicinal surfaces such as the A- and B-stepped (223) and (553) planes, with α = +11.4° and −12.3°, respectively. The curved surface was prepared by several cycles of Ar^+^ sputtering, O_2_ annealing and high-temperature flashes. As described in ref (19), this yields a contaminant-free surface with the expected variation of the step density across the sample.

### Planar Laser-Induced Fluorescence

PLIF was employed to measure the CO_2_ production above the curved catalyst surface.^30,31^ A broadband laser centered at a wavelength of λ = 2.7 μm is used to excite multiple vibrational transitions in the CO_2_ molecules. The most intense fluorescence transition occurs at λ = 4.3 μm, which is collected using a set of lenses and an IR camera. Since the laser is shaped into a sheet by a cylindrical lens, the fluorescence can be measured in 2D across the catalytically active sample.^10^ Each image has a spatial resolution of 30 μm per pixel, and the acquisition rate was 10 frames per second. This yields a time resolution of 1 s after averaging 10 images. The experimental setup is described in more detail in ref (32). During measurements, the sample is placed in a flow reactor with optical access from four sides and heated using an inert boron nitride heater, where we measure the temperature according to ref (33).

### Near-Ambient-Pressure X-ray Photoemission Spectroscopy

**Figure 2:**
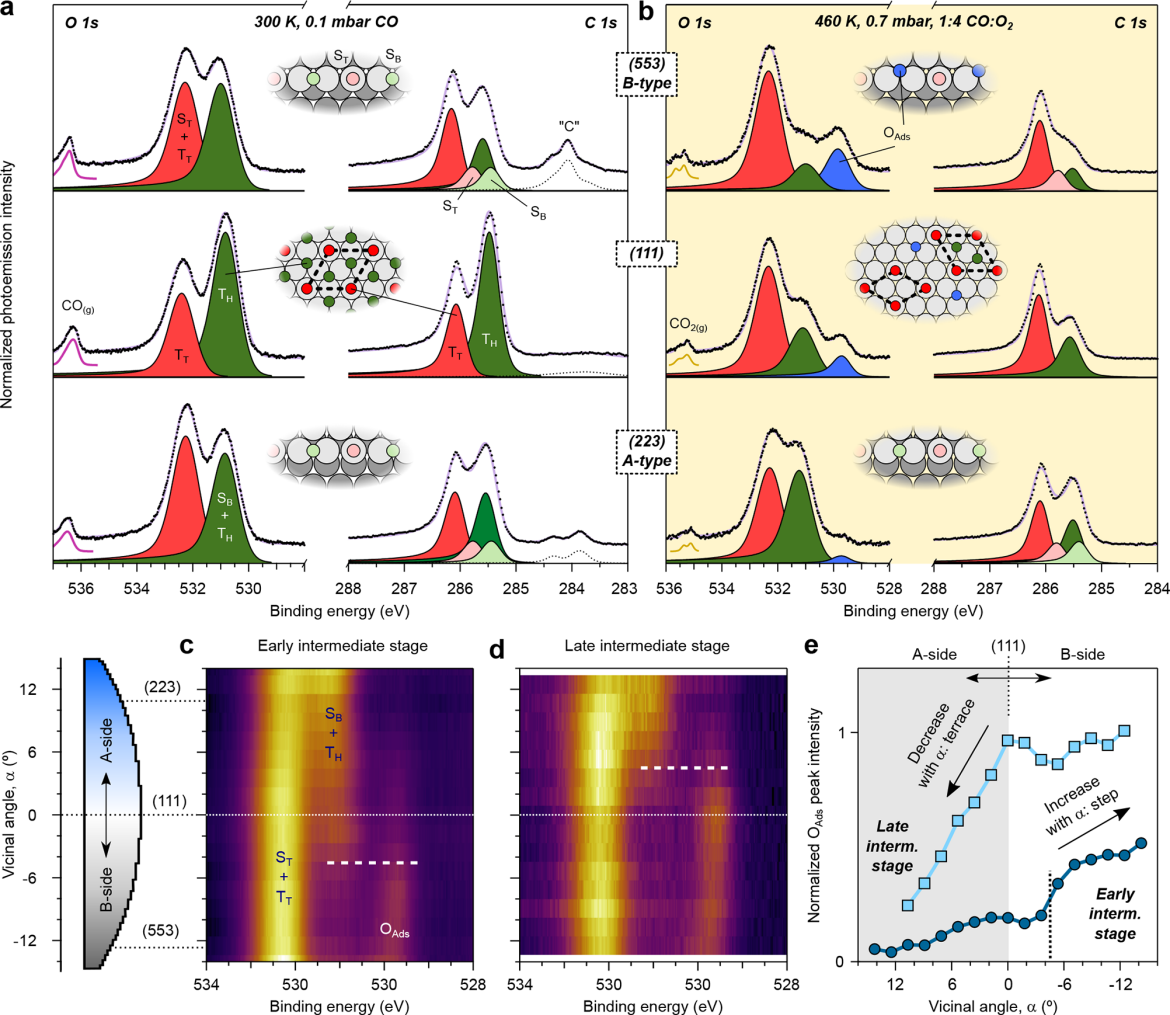
O 1s (*hν* = 650 eV) and C 1s (*hν* = 400 eV) spectra at (a) 0.1 mbar CO at 300 K (far from light-off) and (b) 0.7 mbar in a 1:4 CO:O_2_ gas mixture at 460 K, right after the first ignition step. Top, middle, and bottom rows correspond to the data acquired at the (553), (111), and (223) planes, respectively. T_T_, T_H_, S_T_ and S_B_ refer to CO molecules anchored in Terrace-Top, Terrace-Hollow, Step-Top and Step-Bridge sites. O_Ads_ and “C” stand for atomic oxygen at fcc sites and amorphous carbon, respectively. Insets illustrate the surface composition of the facets in each case. (c, d) O 1s photoemission intensity scans along the curved Rh surface (α-scan) for (c) the 1:4 mixture of panel (b) (0.7 mbar, 460 K), and (d) for a 1:1 CO:O_2_ gas mixture (1.0 mbar, ∼530 K). They respectively reflect an early and a late intermediate ignition stage. (e) O_Ads_ peak area across the *c*-Rh(111) sample, as determined from line fit analysis of the spectra in (c, d) α-scans (see also Figure S2 in the Supporting Information). NAP-XPS data in panels (b) and(c) have been acquired at NSLS-II with a flux smaller than 1 mL/min, and in panel (d) at ALBA with 2.5 mL/min flux.

**Figure 3:**
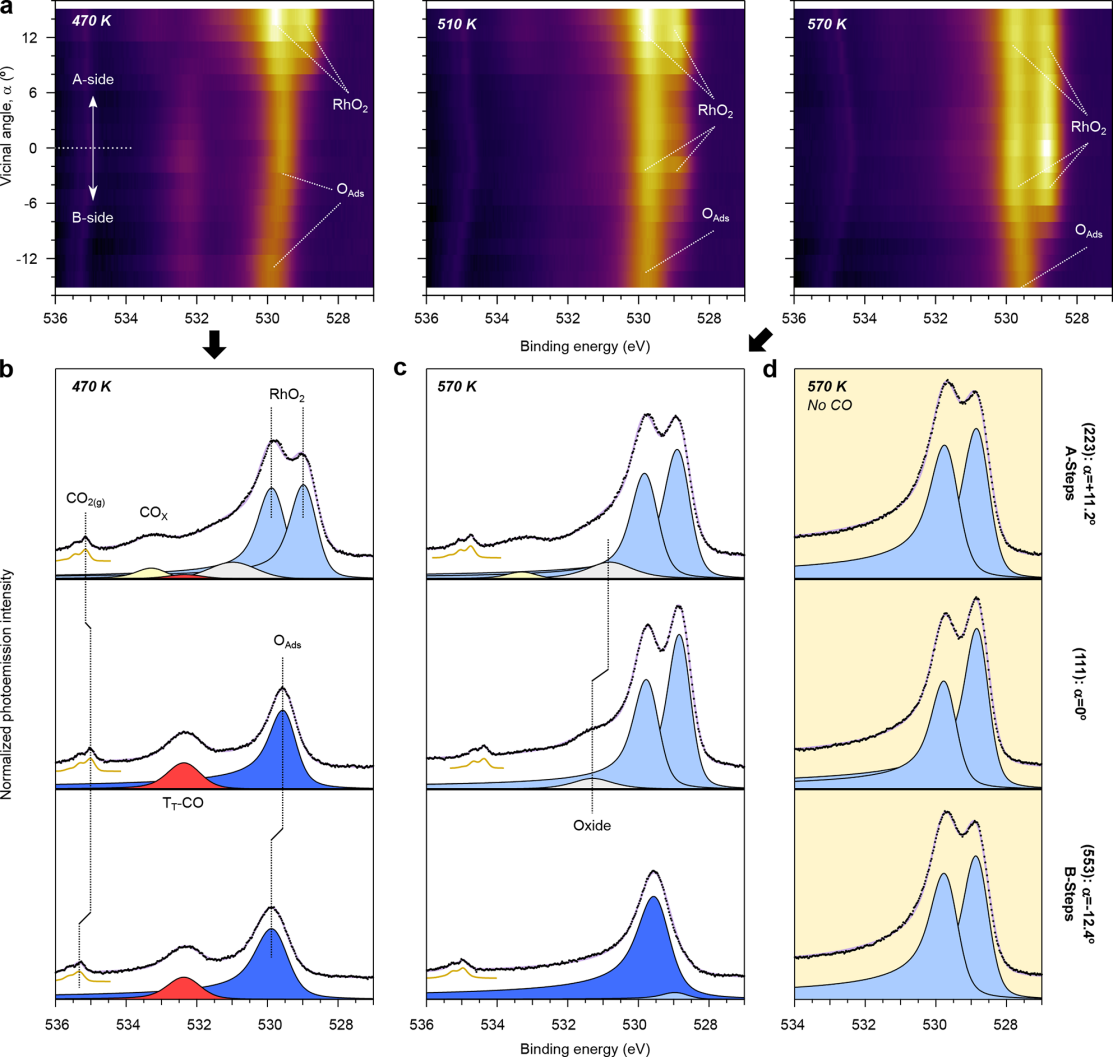
(a) O 1s α-scans acquired at 470, 510, and 570 K under a 1:4 CO:O_2_, 0.7 mbar gas mixture. Spectra were taken at *hν* = 650 eV at NSLS-II, after those shown in Figures 2b and 2c. (b, c) Individual fitted spectra of the (223), (111), and (553) surfaces at 470 and 570 K under reaction conditions, as well as at (d) 570 K after pumping the CO (light-beige panel). O_Ads_, RhO_2_, and Oxide stand for chemisorbed atomic O, surface oxide trilayers, and an uncharacterized Rh oxide, while CO molecules anchored to Terrace-Top sites and carbonate/carboxyl species are denoted as T_T_ and CO_X_, respectively. Vertical dashed lines are included in panels (b) and (c) to illustrate the shift of Oxide, O_Ads_, and CO_2_(g) with α. The latter is caused by a varying work-function across the curved crystal,^9^ which, in turn, reflects the strong differences in the local surface composition and structure.

The NAP-XPS experiments were conducted at the In Situ and Operando Soft X-ray Spectroscopy (IOS, 23-ID-2) beamline of NSLS-II synchrotron, using normal emission geometry and 50° incidence angle, with respect to the (111) plane,^34^ and Circe beamline of the ALBA synchrotron, using a 55° emission angle and a 15° incidence angle, with respect to the (111) plane.^35^ The experiments at NSLS-II were performed using an inert boron nitride heater, and the chamber was pumped through the electron analyzer nozzle (<1 mL/min). For the experiments at ALBA, an encapsulated Pt filament was used as a heater, and the flux was ∼2.5 mL/min. Variations in the ignition temperature are expected due to the differences in the reactants ratio, heater, flow, and thermocouple position. Accordingly, the temperature for the 1:1 CO:O_2_ ratio NAP-XPS experiment shown in Figures 2d and 2e, as well as Figure S3b in the Supporting Information, is determined with ±15 K accuracy. No time evolution of the spectra was observed during the α-scans shown in Figures 2 and 3 (∼2–3 h). In order to compare O_Ads_ during the early and late intermediate stages shown in Figure 2e, spectra at NSLS-II and ALBA were normalized to the height of T_T_-CO at the (111) plane. Signal-to-noise is lower in the ALBA experiments due to a faster acquisition mode, compared to the spectra measured at NSLS-II.

### Peak Fitting of the Photoemission Spectra

Peak fitting was performed with Python’s lmfit package.^36^ Surface components were fitted to Doniac-Šunjić lineshapes^37^ convoluted with a Gaussian profile, while Voigt profiles were considered for gas-phase peaks. Using the spectra at the (111) plane at 0.1 mbar CO at 300 K as a reference, chemisorbed CO peaks were constrained to have a similar width and asymmetry, while subtle changes in position (<50 meV) were allowed at the different temperatures to improve the fit. During the active stage, the asymmetry of the O-species was alike, although the high binding energy component of the RhO_2_ doublet was wider than the other contributions. After pumping the CO from the chamber, the RhO_2_ doublet become significantly more asymmetric, compared to reaction conditions.

## Results

### Asymmetric Light-off at A- and B-Steps

We first investigate the CO ignition across the curved Rh(111) sample [*c*-Rh(111)] by PLIF, which allows a spatial and temporal imaging of the CO_2_ production above the curved surface.^10,11^ The *c*-Rh(111) crystal is identical to that described in ref (19), and appears sketched at the bottom of Figure 1a. It provides a smooth variation of the density of steps as one leaves the Rh(111) plane located at the center. At one side, one finds A-type close-packed steps (square, 100 microfacet), while B-Steps (triangular, 111 microfacet) are encountered at the other side. The step density is directly related to the vicinal angle α.^38^ With this particular sample design, one can probe up to α = ±15°, allowing to reach the densely stepped (223) and (553) planes at each of the sample edges. Both (223) and (553) facets feature 5-atom-wide terraces (corner atoms included) separated by either A- or B-type monatomic steps, respectively. For convenience, we use α > 0 for A-Steps and α < 0 for B-Steps.

In PLIF experiments the clean c-Rh(111) sample was exposed to 24 mbar of a 1:5 CO:O_2_ gas mixture and subsequently heated to trigger the reaction. Argon was added as a carrier gas in order to reach a total flow of 0.1 l/min and a total pressure of 150 mbar. In addition, a quadrupole mass spectrometer (QMS) was employed to monitor the gases in the outlet of the cell. CO_2_ PLIF snapshots were continuously taken at different temperatures along the curved surface to track the CO ignition (see Figure 1a). At 485 K, no CO_2_ production is observed due to the CO poisoning of the entire surface. The CO_2_ cloud arises at 498 K at α ≈ – 5° in the B-side of the crystal (tick mark). At 501 K, the CO_2_ cloud extends toward the (111) plane located at the center, and at higher temperatures (503 K) it is detected above the entire *c*-Rh(111) sample. The PLIF images reveal an earlier ignition of B-type Rh(111) vicinals, followed by a progressive extension of the reaction toward the (111) center and the A-side. The activation of the entire c-Rh(111) sample marks the transition to the complete active stage. A somewhat different asymmetric ignition is observed in Pd,^10^ while A-type and B-type surfaces ignite at the same temperature in Pt.^11^

The A-B asymmetry noted in PLIF is mirrored in the simultaneously acquired QMS signal, which, in turn, reveals a two-step ignition process. In Figure 1b we display the CO_2_ QMS intensity during separate ignition cycles with different CO:O_2_ gas ratios. Heating ramp, total pressure and total flow were kept constant. Looking to all curves, we immediately note the increase of the ignition temperature with the CO content, which is a well-known phenomenon.^1^ Considering the 1:5 CO:O_2_ mixture of Figure 1a, we observe that the CO_2_ signal steadily increases as the sample is heated, but it steeply boosts at ∼488 and 501 K. PLIF images in Figure 1a allow one to correlate these steps with the two abrupt ignition events occurring at the B- (*T*_*i*,B_, 488 K) and A-sides (*T*_*i*,A_, 501 K) of the crystal, marking a Δ*T*_AB_ of 13K. The minimum Δ*T*_AB_ gap occurs at the 1:1 CO:O_2_ pressure ratio, and then becomes larger with both the amount of CO or O_2_ in the gas mixture. As we shall discuss below, such processes are the successive activation of B- and A-type atomic steps, separated by the ignition of the (111) terraces in the *T*_*i*,B_-*T*_*i*,A_ temperature range, which we call the intermediate stage. The CO:O_2_ gas ratio strongly influences both the ignition temperature and the relative CO coverage at terraces and steps, and hence the Δ*T*_AB_ gap is heavily dependent on it.

### Evolution of Chemical Species during Light-off

NAP-XPS experiments were performed to explore the chemical species involved in the two-step ignition of the c-Rh(111) sample. For a quantitative characterization of the CO poisoning layer, it is useful to examine the CO adsorption alone, and compare with reference chemisorption experiments performed at low pressures.^19^ To this aim, we exposed the c-Rh(111) crystal to 0.1 mbar CO at 300 K. Spectra acquired at three relevant Rh facets, namely, the (111), A-type (223) and B-type (553) surfaces, are shown in Figure 2a.

At the (111) plane, two well-resolved peaks are observed in both the O 1s and C 1s core level regions. We assign them to CO adsorbed in top (T_T_, 532.4 and 286.1 eV) and hollow (T_H_, 530.8 and 285.5 eV) terrace sites, respectively.^14,15,39^ The intensity ratio T_H_/T_T_ is close to 2, pointing toward the arrangement of the CO molecules in the (2 × 2)-3CO superstructure, with 0.75 ML (ML = monolayer, adsorbed molecules per substrate atom).^14−16^ In the stepped (553) and (223) (top and bottom rows of Figure 2a, respectively), two more features are resolved in the C 1s region. They correspond to CO molecules adsorbed in top (S_T_, 285.8 eV) and bridge (S_B_ 285.4 eV) positions at steps, and as previously reported they equally cover the surface.^18^ Taking into account the 0.75 ML CO coverage at the (111) plane, one can calculate the S_T_- and S_B_-CO coverages, resulting in 0.07 ML each. These values agree well with the saturation model proposed in an earlier publication (1 CO molecule in S_T_ and S_B_ sites per 3 Rh atoms^19^). The CO saturation structure of the aforementioned surfaces are shown in the insets of Figure 2a.

The intensity of the T_H_-CO peak decreases from the (111) surface to the (223) and (553) planes, while that of T_T_-CO does the reverse. This is explained by considering a transition of some CO molecules from T_H_ to T_T_ sites, as T_H_-CO is known to become less favorable as the step density increases and the terraces narrow.^40^ Step-related CO contributions cannot be resolved in the O 1s region,^39^ hence the peaks are renamed as (S_B_ + T_H_) and (S_T_ + T_T_). The CO_(g)_ emission peaks at 536.5 eV. Residual amorphous carbon (“C”, at 284 eV) at the stepped surfaces may either arise from the CO dissociation or adsorption of residual hydrocarbons.

Next, we proceed to activate the catalytic CO oxidation by adding O_2_ to the mixture and then increasing the temperature. The mixture is fixed to a CO:O_2_ ratio of 1:4 with a total pressure of 0.7 mbar. The temperature is increased until a significant increase of the CO_2_ production is detected at 460 K. At this temperature, the CO_2_ turnover is roughly half of that achieved upon full sample activation, which indicates that we lie between the first and the second ignition steps, i.e., in the intermediate stage. Spectra are shown in Figure 2b. Measurements at temperatures below the first ignition step are discussed in Figure S1 in the Supporting Information. Note that absolute temperature values may differ from those in PLIF experiments, which were performed under very different conditions, particularly flux.^11^ Because of the high turnover, CO(g) coexists now with CO_2_(g) in the spectra (shown in Figure S2 in the Supporting Information).

O 1s and C 1s spectra have significantly changed in the (111) plane, compared to 300 K. CO anchored at T_H_ sites has reduced its intensity by more than 60% at 460 K (middle row of Figure 2b). The reason for this is that the CO adsorption energy is lower in T_T_ sites, compared to T_H_ sites.^14,15^ An extra contribution close to 529.5 eV is also detected, which is ascribed to chemisorbed atomic oxygen (O_Ads_) at hollow fcc sites.^20,21,41^ The desorption of T_H_-CO creates empty surface sites, which allow the O_2_ dissociative adsorption to occur and explains the presence of O_Ads_. Furthermore, the decrease in T_H_-CO also accounts for the slight increase of T_T_-CO, since a portion of the remaining adsorbed CO molecules may rearrange into  R30°-CO domains. These feature a larger T_T_-CO coverage than the (2 × 2)-3CO superstructure, hence explaining its growth just before the ignition.

The C 1s spectrum at the (223) plane at 460 K (top row of Figure 2b) is quite similar to that acquired at 300 K in the CO atmosphere alone: the step peaks (S_T_- and S_B_-CO) are identical, while both terrace contributions decrease, but particularly T_H_-CO. In contrast, the (553) plane (bottom row of Figure 2b) has evolved substantially: in addition to the large decrease of T_H_-CO (∼70%), the feature related to S_B_-CO has vanished from the C 1s region. Therefore, the photoemission spectra reveal a significant asymmetry after the first ignition step: A-type steps remain close to CO saturation, while CO has significantly desorbed from B-steps. Meanwhile, a significant amount of CO has also desorbed from T_H_ sites everywhere. Since desorption is the opposite to adsorption, these results agree with the fact that sticking at T_H_ and S_B_ sites occurs at a less efficient rate, compared to T_T_ and S_T_ positions at Rh vicinals,^17−19^ while CO adsorption at T_H_ and S_B_ sites is faster at A-type Rh steps.^19^

CO-related peaks show the same effect in the O 1s spectra, i.e., nearly the saturation intensity at A-steps and a strong decay at B-steps. Here, we focus on the O_Ads_ peak. Its intensity is roughly half of the T_H_-CO feature at the (111) plane, and it is very small at the (223) surface. This points toward adsorption of oxygen at terrace fcc terrace sites at (111) and (223) facets. In contrast, O_Ads_ is almost double that of the (S_B_ + T_H_) contribution in the (553) plane, and it is also larger than O_Ads_ at the (111) surface. We conclude that, together with a minor adsorption at terraces, oxygen adsorbs majorly at fcc sites of B-stepped edges. In fact, the C 1s analysis reveals partially CO-depleted B-steps, which allow oxygen to accumulate and start the CO oxidation earlier than at the CO-poisoned A-type steps, as observed in PLIF. The chemical composition of each surface is illustrated in the insets in Figures 2a and 2b.

The smooth variation of the surface orientation of curved crystals allows us to finely investigate the surface species as a function of the vicinal angle α. By scanning the curved surface with the small synchrotron ligth beam, one can create photoemission intensity maps that image the distribution of adsorbates across the different vicinal planes.^9,11^ In Figure 2c, we show the O 1s α-scan for the 1:4 CO:O_2_ mixture of Figure 2b. The (S_B_ + T_H_) feature steadily grows from the center to the top of the image (A-side, α > 0), while it decreases from the center to the bottom (B-side, α < 0). This confirms that CO adsorbs to S_B_ sites solely at A-steps, and not at B-steps. O_Ads_ follows the reverse behavior, hence oxygen sticks to the CO-depleted B-steps and not to the CO-poisoned A-steps.

The dashed white line in Figure 2c marks the crossover from a (S_B_ + T_H_)-covered surface to a O_Ads_-covered one at α ≈ −5°. This α angle is coincident with the center of the emerging CO_2_ cloud in the 498 K PLIF image of Figure 1b, which may simply reveal that the α-scan in Figure 2c characterizes an “early” intermediate stage, immediately after the first ignition step. In fact, altering the reaction conditions is possible to reach a “late” intermediate stage, closer to the second ignition step, where the intensity and distribution of (S_B_ + T_H_) and O_Ads_ species across the c-Rh(111) surface are markedly different. This is shown in the α-scan of Figure 2d, which has been obtained for a 1:1 CO:O_2_ mixture at ∼530 K. We immediately notice that the (S_B_ + T_H_)-O_Ads_ crossover point has shifted toward the A-side the crystal, as it happens with the CO_2_ cloud in PLIF as the ignition progresses (Figure 1b).

A quantitative comparison of the O_Ads_ intensity between Figures 2c and 2d surface allows deeper insights into the evolving chemical composition of the intermediate stage. This analysis is shown in Figure 2e, where data points are determined from the peak fit of individual spectra in the respective α-scans (selected spectra are shown in Figure S2). Generally, there is a larger O_Ads_ signal in the 1:1 case, as expected for a surface that is more depleted of CO at a more advanced ignition stage at a higher temperature. The α-dependent trends are also different in both cases, revealing a different O_Ads_ filling of terrace and step sites in each case. To understand the intensity variation in these curves one must note that, because of the lateral extension of the steps, terrace and step signals in a curved surface are expected to respectively decrease and increase as a function of α.^38^ In the 1:1 case, the O_Ads_ intensity is virtually constant at the B-side, indicating that both steps and terraces are equally occupied by oxygen. In contrast, at the A-side, where steps remain CO-poisoned, the intensity decreases linearly as a function of α, reflecting the decaying contribution of O_Ads_-covered terraces to the total peak emission. In the 1:4 case, the linear decrease in the A-side also reveals exclusive, but minor O_Ads_ occupation of terraces. In contrast, the B-side exhibits a α-dependent increase of the O_Ads_ intensity, which reflects the oxygen adsorption of B-step sites.

In summary, the surface chemistry analysis of Figure 2 allows one to explain the two-step ignition of the c-Rh(111) surface discussed in Figure 1. Abrupt changes in the CO_2_ turnover are due to the sequential activation of B-steps at *T*_*i*,B_, due to CO desorption from S_B_ sites and O_Ads_ adsorption, and afterward A-steps at *T*_*i*,A_. Activation of (111) terraces appears to be progressive and occurs at intermediate temperatures, when CO desorption allows O_Ads_ occupation of T_H_ sites. Although CO desorption from T_T_ sites from both sides of the crystal may be most significant at *T*_*i*,A_, a sizable amount remains at higher temperatures in nonoxidized facets, as we discuss next.

### Chemical Species in the Active Stage

Further heating of the 1:4 CO:O_2_ mixture to 470 K triggers the reaction. Most of the CO leaves the surface, CO(g) vanishes from the spectra and the maximum CO_2_ production is reached, marking the activation of the entire sample. Figure 3a shows O 1s α-scans acquired at different temperatures above the ignition point (470, 510, and 570 K), while Figures 3b–d display fitted spectra for selected positions across the curved crystal. They unveil, in the most direct way, a striking step-related trend in surface oxidation during the active stage: the progressive formation of O–Rh–O (RhO_2_) trilayers from the A-side toward the B-side of the crystal as the temperature is increased. At 470 K, only the spectra at α > 6° (A-side of the sample) feature a pair of peaks at 529.5 and 528.5 eV, while a single contribution is observed in the rest of the crystal. Such doublet represents the superficial and interstitial O-layers of the RhO_2_ surface oxide trilayer.^20,21^ This means that, at this temperature, a O–Rh–O trilayer forms at densely stepped surfaces with A-steps (two well-defined features at 529.5 and 528.5 eV), while oxygen remains in its more active chemisorbed form at (111) terraces and B-steps (single peak at ∼529 eV). At the B-side, O_Ads_ exhibits an α-dependent binding energy shift, as well as a peak broadening toward the (553) edge of the sample (Figure 3b), which likely reflects the presence of terrace and step species that cannot be resolved.^20,21^ The scenario is slightly different after reaching 510 K: the RhO_2_ doublet arises at the (111) plane, indicating that terraces start to form O–Rh–O trilayers as the temperature of the sample increases. The oxidation of the terraces is further enhanced after reaching 570 K, and the RhO_2_ signal extends to the B-side up to α = −6°. RhO_2_ steadily decreases in the range of −6° < α < −10°, while beyond α = −10°, only O_Ads_ is detected. At 570 K (Figure 3c), the oxygen remains chemisorbed at B-steps under reaction conditions, while the other facets of the sample have fully developed RhO_2_ trilayers.

Important details inside the α-scan images are better observed in the single spectra shown in Figures 3b and 3c. Similarly to Pd,^9^ Ir,^42^ and Ru,^43^ and in clear contrast to Pt,^42,44^ a sizable amount of CO can still be detected at both the (111) and (553) Rh facets after the ignition. The significantly smaller CO peak at the (223) plane reflects the fact that RhO_2_ quenches the CO adsorption.^20^ An additional feature arises at 533.3 eV at the (223) surface. As judged by its binding energy, we assign this peak to carbonates/carboxyls (CO_X_)^45^ anchored exclusively at the A-steps. Small extra contributions [531.0 eV at the (111) plane, 530.5 eV at the (223) plane] are observed in parallel to the surface oxide doublet, which we attribute to a different Rh oxide. This feature vanishes once the CO is removed from the gas mixture, suggesting a reaction-stabilized oxide^46^ or reaction intermediate.^44^

Under the oxidative reaction conditions of Figure 3c, O_Ads_ can only be removed from the B-edge of the sample by closing the CO valve, which leads to a progressive pumping of CO from the chamber, and to a pure O_2_ atmosphere within minutes. The resulting spectra are shown in Figure 3d. The surface oxide covers the entire sample, but peaks become more asymmetric. This points to the buildup of additional, unresolved oxidic species, which would contribute to the high binding energy tail of the RhO_2_ doublet. Similar spectra are obtained in the reference oxidation shown in Figure S3 in the Supporting Information. The fact that removing the CO from the gas feed is required to further oxidize B-stepped surfaces is very meaningful. It reveals a larger CO oxidation rate at B-Steps, compared to A-Steps, since the formation of the surface oxide would be hindered if the CO oxidation kinetics are faster than those of the metal oxidation.^47,48^ Surface reduction kinetics are further accelerated, and no surface oxide is formed if the CO:O_2_ ratio is closer to stoichiometry (1:1 pressure ratio, see Figure S3). Although faster CO oxidation kinetics are expected for B-steps rather than A-steps, we could not estimate this effect with the data available. After the ignition, the reaction reaches the mass-transfer limit (MLT), where the CO_2_ cloud blocks the diffusion of reactants toward the Rh surface and limits the reaction rate.^49^ As discussed in Figure S4 in the Supporting Information, the CO_2(g)_ peak exhibits the same random intensity variation as the O_2_(g) feature across the *c*-Rh(111) surface. This indicates that, because of insufficient pumping, the CO_2_ cloud equally covers the sample, and hence differences in turnover frequencies cannot be estimated.

Klikovits and co-workers studied the oxidation of A- and B-type Rh(111) steps, observing closed and open oxide structures for A- and B-type steps, respectively.^50^ The open oxide would be tentatively easier to reduce than the closed one, simply explaining the larger activity of B-steps over A-steps for the CO oxidation that we postulate. In any case, RhO_2_ is known to be less active toward CO oxidation than O_Ads_.^12,28^ On the other hand, Gustafson et al. concluded that specific Rh crystal planes exposed during catalysis will not directly influence the activity.^27^ However, we have shown that there is a clear A-B asymmetry in both the ignition temperature and composition of the active stage of the CO oxidation under the very same experimental conditions. Finally, we must not discard faceting of any of the aforementioned surfaces, since Rh vicinals are known to undergo faceting under oxidative^21,22^ and CO oxidation conditions.^23^ This calls for a combined effort for the simultaneous probing of the chemical species and surface structure during the CO oxidation reaction.^51^

## Conclusions

In summary, we have investigated the role of A- and B-type steps during the CO oxidation at millibar pressures using a curved Rh(111) crystal. PLIF images reveal an asymmetric, two-step light-off, where B-type vicinals ignite at lower temperature than A-type surfaces. NAP-XPS points toward a significant CO-depletion and O-accumulation exclusively at B-steps just before the full sample ignition, while A-steps remain CO-poisoned. After the light-off is completed, A-steps readily develop the less-active RhO_2_ trilayers, while oxygen at B-steps remains chemisorbed and no RhO_2_ is formed under reaction conditions. Therefore, we conclude that B-steps are more active toward the CO oxidation than A-steps. Our experiments using curved surfaces emphasize the need of operando studies on the influence of steps and their interplay with other surface sites in chemical reactions.

## Supporting Information Available

PLIF ignition movie at a CO:O_2_ ratio of 1:5 (MP4)
PLIF ignition movie at a CO:O_2_ ratio of 1:1 (MP4)
PLIF ignition movie at a CO:O_2_ ratio of 2:1 (MP4)
Additional NAP-XPS data during the “C” burning in ALBA, a comparison of the ignition in NSLS-II and ALBA experiments, more details on the active stage during the ALBA measurements and the gas phase variation during the active stage in NSLS-II experiments (PDF)
