# Supplementary material for: Structure Matters: Asymmetric CO Oxidation at Rh Steps with Different Atomic Packing
Source: J Am Chem Soc. 2022 Aug 12;144(33):15363–71. doi: 10.1021/jacs.2c06733 (PMC9413197; doi:10.1021/jacs.2c06733)
Supplement: Supplementary file 4 — ja2c06733_si_004.pdf [file ja2c06733_si_004.pdf]

Structure matters: asymmetric CO oxidation at Rh steps with different atomic packing

Fernando García-Martínez,[*,†,#] Lisa Rämisch,[‡] Khadiza Ali,[¶,@] Iradwikanari Waluyo,[§] Rodrigo Castrillo Bodero,[¶] Sebastian Pfaff,[‡] Ignacio J. Villar-García,[‖] Andrew Leigh Walter,[§] Adrian Hunt,[§] Virginia Pérez-Dieste,[‖] Johan Zetterberg,[‡] Edvin Lundgren,[‡] Frederik Schiller,[¶] and J. Enrique Ortega[*,†,¶,⊥]

†*Departamento Física Aplicada, Universidad del País Vasco, 20018, San Sebastián, Spain*

‡*Department of Physics, Lund University, 221 000, Lund, Sweden*

¶*Centro de Física de Materiales CSIC/UPV-EHU-Materials Physics Center, Manuel Lardizábal 5, San Sebastián, 20018, Spain*

§*National Synchrotron Light Source II, Brookhaven National Laboratory, NY 11973, Upton, USA*

‖*NAPP Station, CIRCE Beamline, ALBA synchrotron, Carrer de la Llum 2-26, Cerdanyola del Vallès, 08290, Spain*

⊥*Donostia International Physics Centre, Manuel Lardizábal 4, San Sebastián, 20018, Spain*

#*Current address: Deutsches Elektronen-Synchrotron DESY, Notkestraße 85, Hamburg, 22607, Germany*

@*Current address: Department of Microtechnology and Nanoscience, Chalmers University of Technology, Chalmersplatsen 4, Götenborg, 41296, Sweden*

E-mail: fernando.garcia-martinez@desy.de; enrique.ortega@ehu.eus

Supporting Information Available

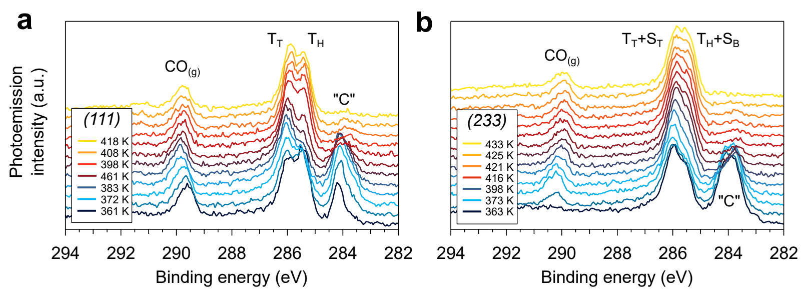

Figure 1: **Separate heating ramps at the (111) and (223) surfaces.** C 1*s* spectra at different temperatures during individual CO ignition experiments at the **a** (111) and **b** (223) planes in a 1.5 mbar, 1:1 CO:O_2 gas ratio mixture. A closer look to the spectra reveals preferred dissociation of CO anchored T_H and S_B positions as the temperature increases, since the intensity of these species decreases while that of “C” grows. The “C” burning begins readily after the CO dissociation, yet no sizeable $CO_{2(g)}$ turnover is observed neither in the XPS nor the QMS. The peaks related to T_H- and S_B-CO partially recover after the “C” vanishes and surface sites become available again. T_T, T_H, S_T and S_B refer to CO molecules in Terrace-Top, Terrace-Hollow, Step-Top and Step-Bridge sites, respectively, while amorphous carbon is labelled as “C”. The experiments were undertaken with a photon energy of 670 eV at ALBA synchrotron, although very similar results were also obtained at NSLS-II when heating from 300 (Fig. 2a) to 460 K (Fig. 2b).

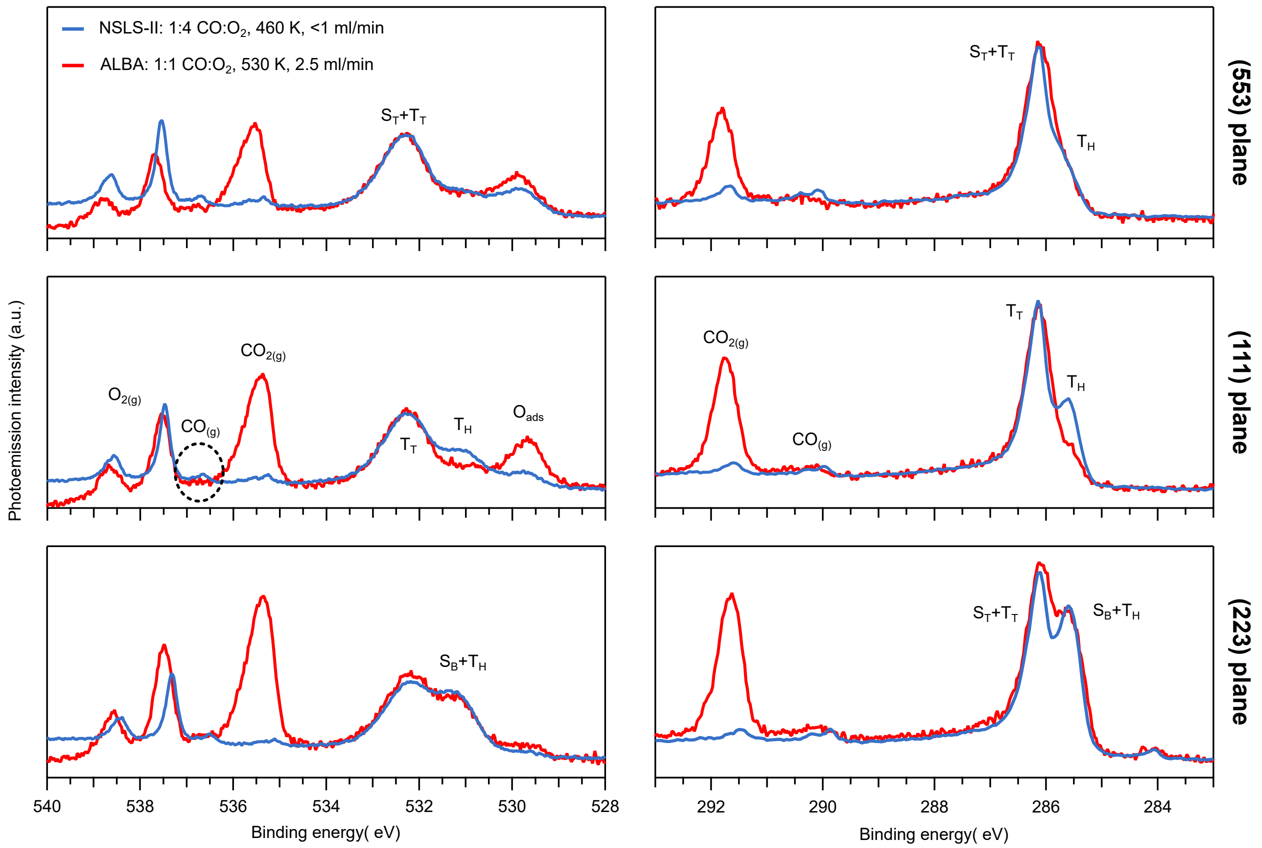

Figure 2: **Differences between 1:1 and 1:4 $CO:O_2$ mixtures just before the complete sample ignition.** O 1*s* and C 1*s* spectra at different surfaces prior to the ignition of the sample. Blue curves correspond to 1:1 $CO:O_2$ ratio (1 mbar, $\sim$ 530 K, 670 eV, 2.5 ml/min flux, experiments performed in ALBA), while red ones to a 1:4 $CO:O_2$ mixture (0.7 mbar, 460 K, 650 eV, $<$ 1 ml/min flux, conducted at NSLS-II). Spectra in both the C 1*s* and O 1*s* regions were normalized so as the height of T_T-CO at the (111) plane is the same. The $CO_{2(g)}$ peak is bigger in 1:1 $CO:O_2$ content, since there is more available CO to react. However, a comparison between gas phase peaks is misleading, since differences in intensity arise due to the geometry of the experimental setup. The build-up of O_{Ads} at the (111) terraces is clearly larger in the 1:1 $CO:O_2$ gas mixture, indicating that we are in the late intermediate stage (O accumulation at B-Steps and terraces) for the this case, and in the early intermediate stage (O accumulation mostly at B-Steps) in the 1:4 $CO:O_2$ gas mixture. Note that in ALBA there is less CO at the terraces due to the higher temperature, and hence more sites are available for the O_2 dissociation. Following the text, T_T, T_H, S_T and S_B refer to CO molecules in Terrace-Top, Terrace-Hollow, Step-Top and Step-Bridge sites, respectively. O_{Ads} corresponds to chemisorbed atomic O at *fcc* sites. The shift of the gas lines at each of the Rh facets is related to a different work-function across the curved crystal.[1]

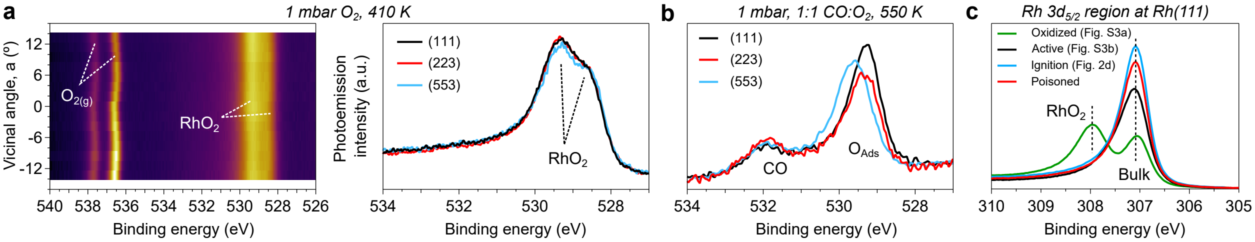

Figure 3: **Additional c-Rh(111) oxidation experiments.** **a** O 1s α-scan at 1 mbar O_2 and 410 K, with selected spectra at the (111), (223) and (553) planes. The characteristic doublet of the surface oxide trilayers (529.5 and 528.5 eV) is easily perceived across the curved surface.[2] This indicates that all Rh facets develop the surface oxide.[2–9] **b** Same surfaces under a 1 mbar, 1:1 CO:O_2 gas mixture at $\sim$ 550 K (active stage of the CO oxidation). Since no doublet related to RhO_2 is observed, the oxygen on the surface remains chemisorbed.[2] This reflects that further oxidation of the surface is quenched as the amount of CO on the gas mixture increases.[10,11] **c** Corresponding Rh 3$d_{5/2}$ region for the (111) plane at different experimental conditions. No surface components are resolved under reaction conditions, possibly due to the relatively high photon energy. Only the RhO_2 signature is observed upon $O_{2(g)}$ exposure. Adsorbed CO molecules, chemisorbed atomic O and the Rh surface oxide are denoted as CO, O_{Ads} and RhO_2, respectively. The experiments were carried out at ALBA synchrotron at a photon energy of 670 eV.

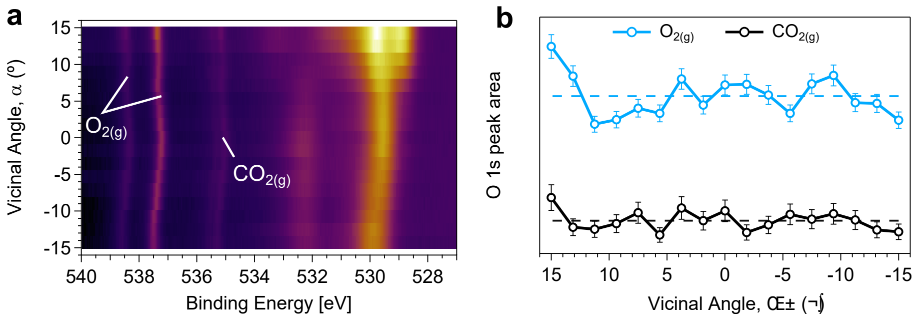

Figure 4: **$O_{2(g)}$ and $CO_{2(g)}$ variation across the surface during the active stage.** **a** Complete O 1*s* α-scan acquired at 470 K, *i.e.* during the active stage of the reaction. Here we include all gas phase lines to illustrate how the reaction has reached Mass-Transfer-Limit (MTL). The other features of the spectra are described in more detail in Fig. 3a. In **b** we show the $O_{2(g)}$ and $CO_{2(g)}$ peaks area across the curved surface. Error bars refer to spectral noise and peak background definition. It does not include intensity variations due to sample positioning (distance between sample and analyzer) in the α-scan, which is known to considerably affect the gas phase features. All in all, the intensity of the $O_{2(g)}$ peak appears to exhibit the same random intensity variations as the $O_{2(g)}$ feature across the c-Rh(111) surface. Since the catalytic activity is different at each point, this indicates an effective accumulation of $CO_{2(g)}$ on top of the surface, *i.e.* the experiment is in MTL. Therefore, the relative turnover frequency variation among different facets cannot be estimated. The photon energy was 650 eV, and the measurements were acquire in a 1:4 CO:O_2, 0.7 mbar gas mixture.

References

(1) Schiller, F.; Ilyn, M.; Pérez-Dieste, V.; Escudero, C.; Huck-Iriart, C.; Ruiz del Arbol, N.; Hagman, B.; Merte, L. R.; Bertram, F.; Shipilin, M.; Blomberg, S.; Gustafson, J.; Lundgren, E.; Ortega, J. E. Catalytic Oxidation of Carbon Monoxide on a Curved Pd Crystal: Spatial Variation of Active and Poisoning Phases in Stationary Conditions. *Journal of the American Chemical Society* **2018**, *140*, 16245–16252.

(2) Lundgren, E.; Gustafson, J.; Resta, A.; Weissenrieder, J.; Mikkelsen, A.; Andersen, J. N.; Köhler, L.; Kresse, G.; Klikovits, J.; Biederman, A.; Schmid, M.; Varga, P. The surface oxide as a source of oxygen on Rh(111). *Journal of Electron Spectroscopy and Related Phenomena* **2005**, *144-147*, 367–372.

(3) Gustafson, J.; Resta, A.; Mikkelsen, A.; Westerström, R.; Andersen, J. N.; Lundgren, E.; Weissenrieder, J.; Schmid, M.; Varga, P.; Kasper, N.; Torrelles, X.; Ferrer, S.; Mittendorfer, F.; Kresse, G. Oxygen-induced step bunching and faceting of Rh(553): Experiment and *ab initio* calculations. *Physical Review B* **2006**, *74*, 35401.

(4) Mittendorfer, F.; Franz, T.; Klikovits, J.; Schmid, M.; Merte, L. R.; Shah Zaman, S.; Varga, P.; Westerström, R.; Resta, A.; Andersen, J. N.; Gustafson, J.; Lundgren, E. Oxygen-Stabilized Rh Adatoms: 0D Oxides on a Vicinal Surface. *The Journal of Physical Chemistry Letters* **2011**, *2*, 2747–2751.

(5) Zhang, C.; Lundgren, E.; Carlsson, P. A.; Balmes, O.; Hellman, A.; Merte, L. R.; Shipilin, M.; Onderwaater, W.; Gustafson, J. Faceting of Rhodium(553) in realistic reaction mixtures of carbon monoxide and oxygen. *Journal of Physical Chemistry C* **2015**, *119*, 11646–11652.

(6) Westerström, R.; Wang, J. G.; Ackermann, M. D.; Gustafson, J.; Resta, A.; Mikkelsen, A.; Andersen, J. N.; Lundgren, E.; Balmes, O.; Torrelles, X.; Frenken, J.

W. M.; Hammer, B. Structure and reactivity of a model catalyst alloy under realistic conditions. *Journal of Physics: Condensed Matter* **2008**, *20*, 184018.

(7) Nolte, P.; Stierle, A.; Jin-Phillipp, N. Y.; Kasper, N.; Schulli, T. U.; Dosch, H. Shape Changes of Supported Rh Nanoparticles During Oxidation and Reduction Cycles. *Science* **2008**, *321*, 1654–1658.

(8) Flege, J. I.; Sutter, P. In situ structural imaging of CO oxidation catalysis on oxidized Rh(111). *Physical Review B - Condensed Matter and Materials Physics* **2008**, *78*, 10–12.

(9) Gustafson, J.; Westerström, R.; Mikkelsen, A.; Torrelles, X.; Balmes, O.; Bovet, N.; Andersen, J. N.; Baddeley, C. J.; Lundgren, E. Sensitivity of catalysis to surface structure: The example of CO oxidation on Rh under realistic conditions. *Physical Review B - Condensed Matter and Materials Physics* **2008**, *78*, 1–6.

(10) Gustafson, J.; Blomberg, S.; Martin, N. M.; Fernandes, V.; Borg, A.; Liu, Z.; Chang, R.; Lundgren, E. A high pressure x-ray photoelectron spectroscopy study of CO oxidation over Rh(100). *Journal of Physics Condensed Matter* **2014**, *26*, 055003.

(11) Gustafson, J.; Westerström, R.; Balmes, O.; Resta, A.; Van Rijn, R.; Torrelles, X.; Herbschleb, C. T.; Frenken, J. W. M.; Lundgren, E. Catalytic activity of the Rh surface oxide: CO oxidation over Rh(111) under realistic conditions. *Journal of Physical Chemistry C* **2010**, *114*, 4580–4583.
